# Administration practices of and adherence to nusinersen in children with spinal muscular atrophy: a multicenter disease registry study in China

**DOI:** 10.1186/s12887-024-05290-0

**Published:** 2025-03-27

**Authors:** Jing Peng, Xiaoli Yao, Rong Luo, Xiuxia Wang, Liwen Wu, Jianmin Zhong, Ruifeng Jin, Xinguo Lu, Jianmin Liang, Siqi Hong, Lin Yang, Xiaoli Zhang, Shanshan Mao, Zhe Tao, Jun Hu, Dan Sun, Hua Wang, Li Zhang, Yanyan Xia, Ken Chen, Yi Wang

**Affiliations:** 1 Department of Pediatrics, Xiangya Hospital of Central South University, Changsha, China; 2 Department of Neurology, The First Affiliated Hospital, Sun Yat-sen University, Guangzhou, China; 3 Department of Pediatric Neurology, West China Second University Hospital, Sichuan University, Chengdu, China; 4 Department of Pediatrics, The Second Hospital of Hebei Medical University, Shijiazhuang, China; 5 Department of Neurology, Hunan Children’s Hospital, Changsha, China; 6 Department of Neurology, Jiangxi Provincial Children’s Hospital, Nanchang, China; 7 Department of Neurology, Children’s Hospital Affiliated to Shandong University, Jinan, China; 8 Department of Neurology, Shenzhen Children’s Hospital, Shenzhen, China; 9 Department of Pediatric Neurology, The First Hospital of Jilin University, Changchun, China; 10 Department of Neurology, Children’s Hospital of Chongqing Medical University, Chongqing, China; 11 Department of Pediatric Internal Medicine, The Second Affiliated Hospital of Xi’An Jiaotong University, Xi’an, China; 12 Department of Pediatric Neurology, The Third Affiliated Hospital of Zhengzhou University, Zhengzhou, China; 13 Department of Neurology, Children’s Hospital, Zhejiang University School of Medicine, National Clinical Research Center for Child Health, Hangzhou, China; 14 Department of Neurology, Dalian Women and Children’s Medical Group, Dalian, China; 15 Department of Pediatric Internal Medicine, Fujian Medical University Union Hospital, Fuzhou, China; 16 Department of Neurology, Wuhan Children’s Hospital, Tongji Medical College, Huazhong University of Science and Technology, Wuhan, China; 17 Department of Pediatric Neurology, Shengjing Hospital of China Medical University, Shenyang, China; 18 Biogen Biotechnology (Shanghai) Co., Ltd, Shanghai, China; 19 Real World Solutions, IQVIA Solutions Enterprise Management Consulting (Shanghai) Co., Ltd, Shanghai, China; 20 Department of Neurology, Children’s Hospital of Fudan University, Shanghai, China

**Keywords:** Nusinersen, Children, Registry, Adherence, Administration practices

## Abstract

**Background:**

Nusinersen was the first approved disease modifying therapy (DMT) for spinal muscular atrophy (SMA). Intrathecal administration of nusinersen enables drug delivery directly to the central nervous system, where the motor neurons are located. Per the package insert, individuals with SMA receive 4 loading doses of nusinersen followed by maintenance doses every 4 months thereafter. The aim of this analysis was to investigate the administration practices of and adherence to nusinersen in Chinese children with SMA.

**Methods:**

Data were analyzed from a longitudinal, multicenter registry enrolling children with 5q-SMA in China. Information on nusinersen administration, including administration date, care setting, use of sedation and general anesthesia, method of administration, and use of imaging guidance before administration, was collected both retrospectively and prospectively. Adherence rate was calculated at dose and participant level. A dose was considered adherent if the inter-dose interval (for dose-level) and interval from the first dose (for participant-level) followed the standard dosing regimen, with a grace period of ± 7 days for Dose 2 to 4 and ± 28 days thereafter.

**Results:**

A total of 385 participants receiving nusinersen with a total of 2,415 doses were included in the study. The median (interquartile range) number of doses administered per participant was 6 (5–7). Over 99% of intrathecal injections were given in an inpatient setting. Only a few (*n* = 3, 0.1%) required general anesthesia, while 9% (*n* = 217) of doses were administered under the use of sedation. Interlaminar lumbar puncture (*n* = 2,407, 99.7%) was the most common method of administration, followed by cervical puncture (*n* = 5, 0.2%) and transforaminal lumbar puncture (*n* = 3, 0.1%). Over 90% of injections did not utilize any imaging guidance prior to administration, with ultrasound (*n* = 142, 5.9%) being the most commonly used imaging guidance. The adherence rate was 95.7% (1,943/2,030) at dose level and 81.0% (312/385) at participant level. The median inter-dose intervals aligned well with the dosing schedule, with 14 days for Doses 2 and 3, 35 days for Dose 4, and 114–124 days for maintenance doses thereafter.

**Conclusions:**

Findings from the analysis demonstrated high real-world adherence to nusinersen in Chinese children with SMA.

**Supplementary Information:**

The online version contains supplementary material available at 10.1186/s12887-024-05290-0.

## Background

Spinal muscular atrophy (SMA) is a neuromuscular hereditary disorder, characterized by muscle atrophy, muscle weakness, progressive loss of motor function, scoliosis, and often early mortality, with a broad spectrum of clinical presentations [1–3]. Subtypes I-IV of SMA are classified based on the age of disease onset and the highest motor milestone achieved. As the first approved disease modifying therapy (DMT) for SMA, nusinersen has dramatically altered the standard of care for patients with the disease. Several clinical trials and observational studies have shown that nusinersen can improve survival and motor function with a favorable safety profile in populations at all ages [4–8].

Nusinersen is indicated for intrathecal use only, with each dose (12 mg/5 mL) administered into the subarachnoid space. The dosing regimen of nusinersen consists of 4 loading doses given over the first 2 months followed by maintenance doses every 4 months [9–11]. Sedation or general anesthesia may be necessary depending on the clinical condition, age and cooperation of the patient. Ultrasound or other imaging techniques may be used to guide administration, particularly in younger patients or in patients with significant spinal deformities or the presence of spondylodesis [9]. From previous studies, an overall high technical success rate in nusinersen administration with conventional lumbar puncture (LP) has been observed. In certain circumstances, such as patients with scoliosis or prior spinal surgery, the complex anatomy may make it more challenging to perform conventional LP, and alternative approaches and/or imaging guidance may be needed in these settings, as has been reported in literature [12–15].

Due to nusinersen’s route of administration and dosing regimen, patients and/or their caregivers need to travel to hospitals and coordinate with physicians’ schedules to receive treatment [16, 17]. Prior studies demonstrated varying adherence rates to nusinersen [18–21]. These studies were mostly conducted in United States (US) with small sample sizes. Real-world studies on administration practices of and adherence rate to nusinersen are lacking in non-US settings.

A national SMA disease registry specifically targeting Chinese individuals aged < 18 years with 5q-SMA was established in 2021 [22]. The objective of this analysis was to evaluate administration practices of and adherence to nusinersen in real-world clinical settings in China among children with SMA using data from this registry.

## Methods

A multicenter, longitudinal registry was established in China in 2021 to gather clinical data on children with 5q-SMA [22]. Study sites included general and pediatric hospitals across China. Recruitment began in November 2021. The present analysis used data from the registry to prospectively and retrospectively collect information on baseline characteristics of participants, as well as records of nusinersen doses and administration details at each dose. Index date was set to the date of nusinersen initiation. Baseline data were collected within 30 days prior to the index date. The observation and follow-up period began on April 28^th^, 2019, when nusinersen was first launched in China, and ended on May 12^th^, 2023, the data transfer date for the present analysis.

### Participants

To be eligible for inclusion in this registry, participants must meet the following criteria: (1) Ability of the participant and/or his/her legally authorized representative (such as parent or legal guardian) to understand the purpose and risks of the study, to provide informed consent, and to authorize the use of confidential health information in accordance with national and local privacy regulations; (2) Genetically confirmed 5q-SMA; (3) Age < 18 years at registry enrollment.

Participants starting nusinersen treatment during the study period regardless of the duration of treatment, dosing frequency, or whether being symptomatic at nusinersen initiation, were included in this analysis.

### Study variables and outcomes

Baseline demographic and clinical characteristics were collected and evaluated in this analysis, including age, sex, SMA type, diagnosis of scoliosis, surgery for scoliosis, wheelchair use and motor function (measured by World Health Organization (WHO) Motor Milestones Assessment [23] among participants aged < 5 years, Children’s Hospital of Philadelphia Infant Test of Neuromuscular Disorders (CHOP-INTEND) [24] among type I participants, Hammersmith Functional Motor Scale–Expanded (HFMSE) [25] among type II and III participants, Hammersmith Infant Neurological Exam–Part 2 (HINE-2) [26] among type I participants, and Revised Upper Limb Module (RULM) [27] among type II and III participants). Nusinersen doses and administration details at each dose, including date of administration, care setting of administration (inpatient or outpatient), method of administration, use of sedation and general anesthesia, and imaging guidance used were also collected.

Adherence rate was calculated in participants with two or more doses, at both dose and participant level. Expected dosing regimen was per package insert [11], with the administration of loading doses on days 0, 14, 28, and 63, followed by maintenance doses at intervals of 4 months. For dose-level adherence, doses were considered adherent if the dose interval between the current and previous dose followed the standard dosing regimen, with a grace period of ± 7 days for loading doses (Dose 2 to 4) and ± 28 days for maintenance dosing. The dose-level adherence rate was defined as the number of doses on time divided by the total number of doses. For participant-level adherence, doses were considered on time if the doses were given per package insert relevant to the date of nusinersen initiation within the grace period of ± 7 days for loading doses (Dose 2 to 4) and ± 28 days for maintenance doses thereafter. Participant-level adherence rate was defined as the percentage of participants who received all doses on time.

### Statistical analysis

Descriptive analyses were used to display the demographic and clinical characteristics of participants and records of dosing details. Continuous variables were summarized by arithmetic mean and standard deviation (SD), or median and interquartile range (IQR). Categorical variables were summarized by the total number of participants and corresponding percentages in each category. In addition, a box plot was created to demonstrate the distribution of dosing intervals at each dose. All analyses were conducted using SAS^®^ software (SAS Institute Inc., Cary, NC; v9.4 or later).

## Results

### Baseline characteristics and nusinersen doses of participants

As of March 2^nd^, 2023, a total of 398 participants were enrolled in the registry. Of them, 385 participants from 18 hospitals received nusinersen, thus were included in this analysis (Table 1). Of 382 participants with known SMA type, 41 participants (10.7%) had SMA type I, 214 (56.0%) had type II, and 127 (33.2%) had type III. A total of 2,415 nusinersen doses were administered. The median (IQR) number of nusinersen doses administered per participant was 6 (5–7), 6 (4–7), 6 (6–7), and 6 (5–7) for participants with all types, type I, II, and III, respectively.

**Table 1:** Baseline demographic and clinical characteristics of participants with SMA

	Total^a^	SMA Type I	SMA Type II	SMA Type III
**Sex**				
Number	385	41	214	127
Male, n(%)	196 (50.9%)	25 (61.0%)	112 (52.3%)	58 (45.7%)
Female, n(%)	189 (49.1%)	16 (39.0%)	102 (47.7%)	69 (54.3%)
**Age at nusinersen initiation**				
Number	385	41	214	127
Median (IQR), months	71 (36–118)	42 (7–54)	62.5 (32–93)	112 (61–154)
< 7 months old, n(%)	13 (3.4%)	10 (24.4%)	1 (0.5%)	0 (0.0%)
≥ 7 months and < 2 years old, n(%)	38 (9.9%)	7 (17.1%)	28 (13.1%)	3 (2.4%)
≥ 2 and < 5 years old, n(%)	106 (27.5%)	14 (34.2%)	68 (31.8%)	23 (18.1%)
≥ 5 and < 8 years old, n(%)	97 (25.2%)	4 (9.8%)	66 (30.8%)	27 (21.3%)
≥ 8 and < 13 years old, n(%)	90 (23.4%)	5 (12.2%)	42 (19.6%)	43 (33.9%)
≥ 13 and < 18 years old, n(%)	41 (10.7%)	1 (2.4%)	9 (4.2%)	31 (24.4%)
**Wheelchair usage**				
Number	264	16	139	108
Yes, n(%)	203 (76.9%)	14 (87.5%)	138 (99.3%)	50 (46.3%)
No, able to walk independently, n(%)	58 (22.0%)	0 (0.0%)	0 (0.0%)	58 (53.7%)
Not applicable, the subject could not sit independently, n(%)	3 (1.1%)	2 (12.5%)	1 (0.7%)	0 (0.0%)
**Diagnosis of scoliosis**				
Number	350	36	192	120
Yes, n(%)	171 (48.9%)	18 (50.0%)	93 (48.4%)	60 (50.0%)
No, n(%)	179 (51.1%)	18 (50.0%)	99 (51.6%)	60 (50.0%)
**Surgery for scoliosis**				
Number	171	18	93	60
Yes, n(%)	2 (1.2%)	0 (0.0%)	0 (0.0%)	2 (3.3%)
No, n(%)	169 (98.8%)	18 (100.0%)	93 (100.0%)	58 (96.7%)
**CHOP-INTEND score**				
Number	/	17	/	/
Mean (SD)	/	24.5 (14.09)	/	/
**HFMSE score**				
Number	/	/	115	91
Mean (SD)	/	/	10.2 (8.89)	36.1 (15.68)
**HINE-2 score**				
Number	/	17	/	/
Mean (SD)	/	3.1 (2.11)	/	/
**RULM**				
Number	/	/	96	77
Mean (SD)	/	/	12.4 (7.82)	27.8 (7.18)

SMA: Spinal Muscular Atrophy; IQR: Interquartile Range; CHOP-INTEND: Children’s Hospital of Philadelphia Infant Test of Neuromuscular Disorders; SD: Standard Deviation; HFMSE: Hammersmith Functional Motor Scale Expanded; HINE-2: Hammersmith Infant Neurological Examination; RULM: Revised Upper Limb Module

a. SMA type was unknown for three participants. Thus, the numbers from columns of SMA Type I, II and III do not sum up to the numbers from column of Total

### Administration practices of nusinersen

Of the 2,415 injections during the analysis period (Table 2), over 99% were given in an inpatient setting. Only 3 injections from 1 single participant required general anesthesia. This participant started the first-dose nusinersen at 1-month old without general anesthesia use, followed by 3 loading doses using general anesthesia. The maintenance doses afterwards were administered with sedation use rather than general anesthesia. The 3 doses with general anesthesia were all administered through interlaminar LP not using any guiding techniques. Overall, fewer than 10% of doses were administered with sedation use. The percentage of doses using sedation slightly varied among SMA types, from 11.6% in type I to 6.2% in type III. Interlaminar LP was the most common method of administration, followed by cervical puncture and transforaminal LP. Over 90% of injections did not utilize any imaging guidance prior to administration. Among all imaging techniques used, ultrasound was most commonly used. Specifically, the 5 doses through cervical puncture came from 1 single participant with scoliosis whose doses were all administered through cervical puncture. The administrations did not require general anesthesia or sedation, and besides the first dose, all doses utilized ultrasound guidance during the administrations. Regarding 2 participants with surgery for scoliosis prior to the initiation of nusinersen, all doses were administered through interlaminar LP, and most doses utilized ultrasound guidance.

**Table 2:** Administration details of nusinersen injections

	Total^a^	SMA Type I	SMA Type II	SMA Type III
**Number of injections**	2,415	233	1,373	788
**Care setting**,** n(%)**				
Inpatient	2,406 (99.6%)	233 (100.0%)	1,367 (99.6%)	785 (99.6%)
Outpatient	9 (0.4%)	0 (0.0%)	6 (0.4%)	3 (0.4%)
Missing^b^	0	0	0	0
**Use of general anesthesia**,** n(%)**				
Yes	3 (0.1%)	0 (0.0%)	0 (0.0%)	0 (0.0%)
No	2,373 (98.6%)	230 (98.7%)	1,349 (98.5%)	776 (99.1%)
Unknown^b^	30 (1.2%)	3 (1.3%)	20 (1.5%)	7 (0.9%)
Missing^b^	9	0	4	5
**Use of sedation**,** n(%)**				
Yes	217 (9.0%)	27 (11.6%)	135 (9.8%)	49 (6.2%)
No	2,146 (88.9%)	198 (85.0%)	1,208 (88.0%)	725 (92.0%)
Unknown^b^	52 (2.2%)	8 (3.4%)	30 (2.2%)	14 (1.8%)
Missing^b^	0	0	0	0
**Method of administration**,** n(%)**				
Interlaminar LP	2,407 (99.7%)	231 (99.1%)	1,367 (99.6%)	788 (100.0%)
Transforaminal LP	3 (0.1%)	2 (0.9%)	1 (0.1%)	0 (0.0%)
Lumbar laminotomy	0 (0.0%)	0 (0.0%)	0 (0.0%)	0 (0.0%)
Cervical puncture	5 (0.2%)	0 (0.0%)	5 (0.4%)	0 (0.0%)
Others	0 (0.0%)	0 (0.0%)	0 (0.0%)	0 (0.0%)
Missing^b^	0	0	0	0
**Imaging techniques used for administration guidance**,** n(%)**				
Ultrasound	142 (5.9%)	4 (1.7%)	69 (5.0%)	69 (8.8%)
Fluoroscopy or radioscopy	1 (0.0%)	1 (0.4%)	0 (0.0%)	0 (0.0%)
CT-scan	1 (0.0%)	0 (0.0%)	1 (0.1%)	0 (0.0%)
Others	2 (0.1%)	0 (0.0%)	2 (0.1%)	0 (0.0%)
Not using imaging techniques	2,204 (91.3%)	223 (95.7%)	1,267 (92.3%)	693 (87.9%)
Unknown^b^	65 (2.7%)	5 (2.1%)	34 (2.5%)	26 (3.3%)
Missing^b^	0	0	0	0

SMA: Spinal Muscular Atrophy; LP: Lumbar Puncture; CT: Computed Tomography

a. A total of 21 injections came from participants with unknown SMA type. Thus, the numbers from columns of SMA Type I, II and III do not sum up to the numbers from column of Total

b. “Unknown” is an option in the case report form of the registry. Records whose certain questions were not answered in the case report form were treated as “Missing” for the corresponding questions and not included in the denominator of percentage calculation

### Adherence

**Fig. 1:**
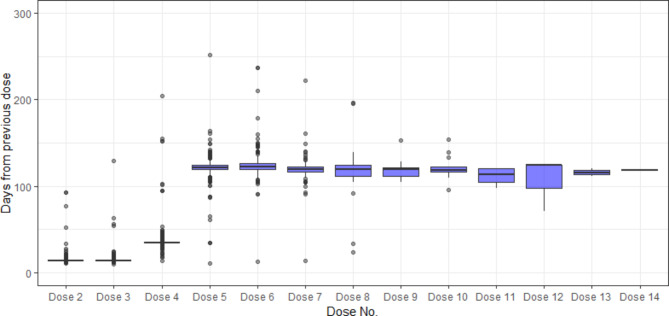
Boxplot of nusinersen inter-dose intervals. The median line represents the median for the inter-dose intervals. The box represents the interquartile range for the inter-dose intervals. The whiskers represent the smallest and largest values within 1.5 times the IQR from the lower and upper quartiles, respectively. For Dose 5, one record with 626 days from previous dose is not displayed in the figure

Distribution of inter-dose intervals at each dose is shown in Fig. 1 and supplementary material. At dose level, the overall adherence rate was 95.7% (1,943/2,030). The median dose intervals aligned well with dosing schedule, with 14 days for Dose 2 and 3, 35 days for Dose 4, and 114–124 days for maintenance doses thereafter. At participant level, the number (percentage) of participants having all nusinersen doses on time was 312/385 (81.0%).

## Discussion

This is the first study to investigate the administration practices and adherence to nusinersen in children with SMA in China, using data from a nationwide registry both prospectively and retrospectively, with a large sample size of participants and recorded nusinersen doses. Since the registry data primarily originated from electronic health records (EHRs), where information on intrathecal procedures was comprehensively captured in the hospital setting, the registry can be considered a reliable resource for studying the intrathecal procedure and drug adherence.

In this study, over 99% of nusinersen doses were administered in an inpatient setting. This contrasts with other studies conducted outside of China, where most nusinersen injections occurred in an outpatient setting [14, 15]. This difference is likely due to the higher reimbursement rates for inpatient versus outpatient medical expenditures in China, leading to a tendency for patients to be hospitalized in many disease settings [28, 29]. Although in most cases nusinersen can be administered without advanced supporting technologies, such as general anesthesia or imaging guidance, the economic consideration may still be essential to drive the selection of care settings.

General anesthesia and sedation increase the procedure time, recovery period, and costs, and impose a heavy burden on the intrathecal procedure. However, in the current study, very few (*n* = 3, 0.1%) LPs required general anesthesia, and this proportion is much lower than reported in other studies [14, 15]. General anesthesia is usually considered for children requiring cervical puncture, those with complex spinal anatomy, and those unable to cooperate [15]. In the current study, the 3 LPs requiring general anesthesia were for 1 participant, most likely because of poor cooperation resulting from young age. We also found that less than 10% of nusinersen doses were administered with sedation use, which is similar to the 9.4% reported by a previous study in the US [14]. The trend of decreasing sedation use from SMA type I to III might be due to the age difference among different SMA types, where older children may be more cooperative and thus less likely require sedation. The low rate of general anesthesia and sedation found in our study suggests low burden of nusinersen administration among children with SMA in China.

Over 99% of intrathecal nusinersen injections were administered via conventional interlaminar LP, and 91% of injections did not utilize any imaging guidance. Conventional interlaminar LP has been shown to be well tolerated in pediatric SMA populations with a high technical success rate [14, 15], and it is usually the route of choice to administer nusinersen in patients without contraindications [30]. In symptomatic patients with scoliosis, spondylodesis, or prior spine surgeries, however, conventional interlaminar LP may be challenging. Thus, alternative approaches to intrathecal access, such as transforaminal LP, cervical puncture, and lumbar laminotomy, are sometimes required [13, 31–33]. Compared with conventional interlaminar LP, these alternative approaches have greater technical difficulty and increased risk of complications [34, 35]. According to a decision-tree algorithm for nusinersen administration [34], conventional interlaminar LP without imaging guidance is considered feasible and safe in non-complex patients, who are defined as those with a Cobb angle of ≤ 50 degrees and without any history of spinal surgery. In this analysis, only 2 participants had a history of spinal surgeries. Most of our participants might be classified as “non-complex” according to the algorithm [34], although Cobb angle results were lacking in this analysis. Among all imaging techniques, ultrasound (*n* = 142, 5.88%) was most commonly used. In complicating circumstances, imaging techniques are often needed to guide the procedure. Among these techniques, ultrasound is usually favored especially in children with SMA, since it does not involve radiation exposure, unlike fluoroscopy or computed tomography (CT) [36].

In previous studies, adherence rates varied significantly partially due to inconsistent definitions of adherence, different data sources, and variations in medical practice across different institutions [18–21]. Prior observational studies utilizing claims databases from US demonstrated varying adherence rates to nusinersen, ranging from 30.0 to 80.5% [18–20], but it is important to note that the inclusion/exclusion criteria used in these studies were likely insufficient to identify the patients with complete nusinersen dosing history. In a study using a combination of US EHRs and claims databases, the adherence rates derived from the EHRs database was higher than that from the claims database (93.9% versus 80.5%), suggesting that claims may not accurately capture the initiation or the complete courses of nusinersen doses, particularly during the loading phase [21]. The registry used in our analysis was primarily sourced from the EHRs from regular clinical practice. Therefore, the registry ensures relatively comprehensive data capture and unbiased evaluation on nusinersen adherence. Overall, we found a high adherence rate of 95.7% (1,943 out of 2,030 doses). The adherence result is particularly notable given that the study period was mostly within the period of the coronavirus disease (COVID)-19 outbreak and repeated widespread lockdown in China, suggesting that patients and their families regarded the nusinersen treatment as a crucial component of SMA disease management. Reasons for the high adherence rate might include: (1) Nusinersen demonstrates effectiveness in improving motor function and reducing lung function decline in individuals with SMA, while maintaining a favorable safety profile with predominantly mild and manageable adverse effects, as shown in other literatures [6, 8, 37]; (2) Starting from 2022, nusinersen was included in the National Reimbursement Drug List in China, resulting in a substantial price reduction [38]. The significant price reduction has greatly increased the accessibility of nusinersen for Chinese patients, enabling them to adhere to a continuous treatment [39].

Of note, low inter-dose intervals (i.e., approximately 2 weeks) were observed for Dose 5 to 7 which are commonly considered as maintenance doses whose inter-dose intervals should be around 4 months. These participants were likely to re-start nusinersen from loading doses for certain reasons, such as gap treatment for a long period. Based on the package insert of nusinersen, patients may require re-loading if gapped ≥ 8 months from last dose [11]. Since the registry could not accurately identify the nusinersen reloading participants, the current calculation of adherence rate assumes that Dose 5 and beyond were all maintenance doses, which would misclassify these re-loading doses from adherent to non-adherent. Given that the dose-level adherence rate in the analysis is particularly as high as 95.7%, the possible misclassification of the adherence of these re-loading doses would not alter the conclusion.

The present analysis has several significant implications for nusinersen treatment of SMA. First, the analysis fills a significant gap in the current knowledge by providing comprehensive data on nusinersen administration and adherence, both locally and globally. With a large sample size of both participants and nusinersen doses, our findings contribute valuable insights into the real-world utilization of nusinersen. Second, the findings from our analysis can serve as a basis for developing guidelines and protocols for the administration of nusinersen across different healthcare settings. For instance, inpatient setting and interlaminar LP without general anesthesia could be included as a standard practice of nusinersen administration by the guidelines and protocols. By establishing standardized practices such as guidelines and institutional protocols, healthcare professionals can ensure consistent and appropriate administration practices of nusinersen, thus ultimately improving patient outcomes. Third, our study suggested that some key information could be included in the education of children with SMA and their parents on the administration of nusinersen to facilitate a better understanding and clearer expectation of the administration process. For instance, it is most likely that they should prepare for a hospitalization at each scheduled administration time, and may encounter irregular method of administration if the patient’s situation is more complex. Peri-procedural pain is not uncommon but should mostly be well-managed without general anesthesia. It is important to strictly follow the instructions from providers before, during and after the procedure to reduce relevant complications. Fourth, this analysis provides essential background information and insights that can inform future studies. By understanding the administration pattern of nusinersen administration, researchers can evaluate the cost-effectiveness of different administration strategies and guide decision-making regarding the most efficient and beneficial use of resources on nusinersen administration. Lastly, the analysis has implications for future research, including but not limited to association between adherence and clinical outcomes, between administration practices and administration-related safety events, and between patient characteristics and administration practices.

There are certain limitations of our study to consider. Firstly, the study design may introduce inherent selection bias due to the nature of a disease registry that includes retrospective visits. The retrospective inclusion of participants on nusinersen and the requirement of informed consent upon registry enrollment may lead to a potential survivor bias towards those with a more favorable prognosis, thus overestimating the adherence rate. Secondly, our data come from an interim analysis of the registry. As a result, the sample sizes for maintenance doses in the later stages (Dose 8 and beyond) were relatively small due to the limited follow-up duration. This limitation will be mitigated as the study progresses and extensive long-term data accumulates. Thirdly, as discussed above, additional loading doses to compensate for delayed maintenance doses were not distinguished from actual delayed or missed doses and were considered as non-adherent doses in the calculation of adherence rates, which would underestimate adherence rates at the dose level.

## Conclusions

This study is the first to comprehensively examine the patterns of nusinersen administration practices and real-world adherence to nusinersen in Chinese children with SMA. The low burden during the administration process and high adherence to nusinersen treatment, even under the period of the COVID-19 pandemic, may indicate nusinersen is generally well-tolerated and patients are committed to continuing their therapy. This study contributes to closing the knowledge gap regarding nusinersen treatment among children with SMA in China and augments the current evidence on nusinersen use and adherence globally. The insights obtained from our study are anticipated to assist in future guideline development and nusinersen administration standardization, ultimately leading to improved care for children with SMA.

## Electronic supplementary material

Below is the link to the electronic supplementary material.

Supplementary Material 1

## Data Availability

Requests from qualified investigators for anonymized data not reported in this article should be submitted to https://vivli.org.

## Abbreviations

CHOP-INTEND: Children’s hospital of philadelphia infant test of neuromuscular disorders
COVID: Coronavirus disease
CT: Computed tomography
DMT: Disease modifying therapy
EHRs: Electronic health records
HFMSE: Hammersmith functional motor scale–expanded
HINE-2: Hammersmith infant neurological exam–part 2
IQR: Interquartile range
LP: Lumbar puncture
RULM: Revised upper limb module
SD: Standard deviation
SMA: Spinal muscular atrophy
SMN: Survival motor neuron
US: United states
WHO: World health organization
