# Supplementary material for: Administration practices of and adherence to nusinersen in children with spinal muscular atrophy: a multicenter disease registry study in China
Source: BMC Pediatr. 2025 Mar 27;25:239. doi: 10.1186/s12887-024-05290-0 (PMC11948911; doi:10.1186/s12887-024-05290-0)
Supplement: Supplementary file 1 — Supplementary Material 1 [file 12887_2024_5290_MOESM1_ESM.docx]

Supplementary Table. Distribution of nusinersen inter-dose intervals in days

|  | **Dose 2** | **Dose 3** | **Dose 4** | **Dose 5** | **Dose 6** | **Dose 7** | **Dose 8** | **Dose 9** | **Dose 10** | **Dose 11** | **Dose 12** | **Dose 13** | **Dose 14** |
| --- | --- | --- | --- | --- | --- | --- | --- | --- | --- | --- | --- | --- | --- |
| Number | 385 | 385 | 378 | 343 | 286 | 167 | 39 | 24 | 12 | 5 | 3 | 2 | 1 |
| Mean (SD) | 15.1 (7.0) | 15.0 (7.1) | 37.4 (15.7) | 121.9 (31.3) | 123.6 (15.7) | 119.2 (13.7) | 117.5 (28.5) | 117.8 (9.8) | 121.4 (14.7) | 111.4 (9.7) | 106.7 (30.9) | 116 .0 (5.7) | 118.0 (N/A) |
| Median | 14 | 14 | 35 | 121 | 122 | 119 | 119 | 119 | 118.5 | 114 | 124 | 116 | 118 |
| Q1, Q3 | 14, 14 | 14, 14 | 35, 36 | 119, 124 | 119, 126 | 117, 122 | 111, 125 | 111.5, 121 | 116, 126 | 105, 120 | 71, 125 | 112, 120 | 118, 118 |
| Min, Max | 11, 93 | 10, 129 | 14, 204 | 11, 626 | 13, 237 | 14, 222 | 24, 196 | 105, 153 | 96, 154 | 98, 120 | 71, 125 | 112, 120 | 118, 118 |
| Expected interval | 14 | 14 | 35 | 120 | 120 | 120 | 120 | 120 | 120 | 120 | 120 | 120 | 120 |
| Number (%) of doses on time^a^ | 375 (97.4%) | 376 (97.7%) | 349 (92.3%) | 327 (95.3%) | 275 (96.2%) | 162 (97.0%) | 35 (89.7%) | 23 (95.8%) | 11  (92.0%) | 5 (100.0%) | 2  (66.7%) | 2 (100.0%) | 1 (100.0%) |

SD: standard deviation

^a^ Using grace period of ±7 days for loading doses (Dose 2 to 4) and ±28 days thereafter from the expected interval.
